# Promoter polymorphisms of *TOP2A* and *ERCC1* genes as predictive factors for chemotherapy in non‐small cell lung cancer patients

**DOI:** 10.1002/cam4.2743

**Published:** 2019-12-03

**Authors:** Anna Grenda, Justyna Błach, Michał Szczyrek, Paweł Krawczyk, Marcin Nicoś, Barbara Kuźnar Kamińska, Monika Jakimiec, Grażyna Balicka, Izabela Chmielewska, Halina Batura‐Gabryel, Marek Sawicki, Janusz Milanowski

**Affiliations:** ^1^ Department of Pneumonology, Oncology and Allergology Medical University of Lublin Lublin Poland; ^2^ Department of Clinical Immunology Medical University of Lublin Lublin Poland; ^3^ Science for Life Laboratory Department of Medical Biochemistry and Biophysics Karolinska Institutet Stockholm Sweden; ^4^ Department of Pulmonology, Allergology and Respiratory Oncology University of Medical Sciences in Poznań Poznań Poland; ^5^ Department of Thoracic Surgery Medical University of Lublin Lublin Poland

**Keywords:** *ERCC1*, non‐small cell lung cancer, single‐nucleotide polymorphisms, *TOP2A*

## Abstract

**Background:**

Topoisomerase 2‐alpha (*TOP2A*) is an enzyme that controls topologic changes in DNA during transcription and replication. *ERCC1* is an enzyme that takes part in DNA repair processes. The purpose of this study was to assess the predictive role of particular single nucleotide polymorphisms (SNPs) in the promoter regions of *TOP2A* and *ERCC1* genes in non‐small cell lung cancer patients (NSCLC) treated with chemotherapy.

**Materials and methods:**

We enrolled 113 NSCLC patients treated in the first line with platinum‐based chemotherapy. Effectiveness was available for 71 patients. DNA was isolated from whole blood using the Qiamp DNA Blood Mini kit (Qiagen). We examined five SNPs: rs11615 (*ERCC1*), rs3212986 (*ERCC1*), rs13695 (*TOP2A*), rs34300454 (*TOP2A*), rs11540720 (*TOP2A*). Quantitative PCR using the TaqMan probe (ThermoFisher) was performed on a Eco Illumina Real‐Time PCR system device (Illumina Inc).

**Results:**

Patients with the A/A genotype in rs11615 of the *ERCC1* gene had significantly longer median progression free survival (PFS) (8.5 months; *P* = .0088). Patients with the C/C genotype in rs3212986 of the *ERCC1* gene had longer median PFS (7 months; *P* = .05). Patients with the C/C genotype in rs34300454 of *TOP2A* gene had significantly higher median PFS (7.5 months; *P* = .0029). Carriers of the C/C genotype in rs34300454 of the *TOP2A* gene had significantly longer median OS (15.5 months; *P* = .0017). Patients with the A/A genotype in rs11615 of the *ERCC1* gene had significantly higher risk of neutropenia (*P* = .0133).

**Conclusions:**

Polymorphisms of the *TOP2A* and *ERCC1* genes may be a predictive factor of toxicities and survival for chemotherapy in NSCLC patients.

## Graphical abstract

The purpose of this study was to assess the predictive role of particular single nucleotide polymorphisms in the promoter regions of topoisomerase 2‐alpha and ERCC1 genes in non‐small cell lung cancer patients. These polymorphism genes may be a predictive factor of toxicities and survival for chemotherapy.

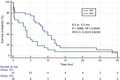

## 1 INTRODUCTION

Lung cancer is the most common cause of death due to malignancies, usually diagnosed in an unresectable, advanced stage. Non‐small cell lung cancer (NSCLC) accounts for 85% of lung cancer patients.^1^, ^2^ Treatment of NSCLC includes surgery, radiotherapy, chemotherapy, immunotherapy (monoclonal antibodies against programmed cell death 1, programmed cell death 1 ligand 1 or cytotoxic T‐lymphocyte associated protein 4) and molecularly targeted therapies (kinases inhibitors of epidermal growth factor receptor, anaplastic lymphoma receptor tyrosine kinase, ROS proto‐oncogene 1, receptor tyrosine kinase or B‐Raf proto‐oncogene, serine/threonine kinase and mitogen‐activated protein kinase kinase 1). Due to the diagnosis of NSCLC in the advanced stage of the disease and rare occurrence of molecular predictive factors, the treatment is limited to chemotherapy or chemoradiotherapy in many patients. Ordinarily, chemotherapy is based on platinum compounds (cisplatin or carboplatin) in combination with a third generation drug, inhibitors of cell divisions (vinorelbine, docetaxel or paclitaxel) or antimetabolites (gemcitabine, pemetrexed).^3^

Platinum compounds form covalent platinum‐DNA adducts and induce damage to the genome. The DNA destruction by chemotherapy is the impulse for cells to repair the lesion and hold the DNA integrity.^4^, ^5^ Vinorelbine is a semisynthetic *vinca* alkaloid. Vinorelbine binds to microtubules, and prevents the formation of the mitotic spindle, resulting in the arrest of tumor cell growth in metaphase during the G2/M phase of the cell cycle. Vinorelbine, as a microtubule destabilizing agent, stimulates microtubule depolymerization.^6^ Taxanes, microtubule‐stabilizing agents, block microtubule polymerization. They are isolated from *Taxus brevifolia* species. Representatives of taxanes available for the treatment of NSCLC patients are paclitaxel and its semisynthetic version—docetaxel.^7^, ^8^ Gemcitabine is a nucleoside analog, which is phosphorylated to gemcitabine triphosphate (dFdCTP) in cells and inhibits DNA replication.^9^, ^10^ Pemetrexed is a folic acid antagonist, which inhibits enzymes participating in the synthesis of purines and pyrimidines, and consequently is a DNA and RNA synthesis inhibitor.^11^

What is wanted for patient nowadays is targeted therapy based on his individual genetic profile. Genetic factors could play a very important role in anticipation and monitoring the response to treatment and survival. Various therapeutic effects are observed using identical treatment protocols, which may be caused by individual variations in the response to DNA damage, and the sensitivity to chemotherapy.^12^ The main contributions to DNA repair mechanisms are nucleotide excision (NER) and base excision repair, DNA mismatch repair, and single‐strand break repair.^13^ Cancer cells use the DNA's ability to repair to oppose the effects of chemotherapy. Nucleotide excision recognizes damage that interferes with the structure of the double helix. Nucleotide excision action consists of local opening of the DNA helix, excising the damage and filling the fissure.^14^ The NER process contains various stages and engages many different proteins, including *ERCC1* (excision repair cross‐complementation group 1).^15^ The ERCC1 endonuclease incises the damaged DNA strand on the 5′ side of the lesion. The ERCC1 nuclease also functions in pathways to repair double‐strand breaks in DNA, and in the repair of crosslink damage that harmfully links the two DNA strands. Base excision repair is the mechanism of correcting DNA damage by removing single DNA base damage during DNA replication.^16^, ^17^

TOP2A (topoisomerase 2‐alpha) is an enzyme that controls topological changes in DNA, segregation of newly replicated chromosomes, condensation and chromosome formation. The inhibition of its activity leads to the formation of bonds within the DNA strand, which results in blocking transcription and translation.^18^, ^19^

Differentiations in genes encoding repair enzymes may have impact on DNA repair during cancer treatment. Single nucleotide polymorphisms (SNPs) in particular genes can predispose to special responses to chemotherapy. This study reviewed the predictive values of SNPs in promoter regions of *TOP2A* and *ERCC1* genes. Based on the above SNPs functions, they can be considered as biomarkers useful for prediction of chemotherapy effectiveness and survival in different cancers, including NSCLC.

The reason for the selection of studied SNPs in *ERCC1* and *TOP2A* genes was associated with the function of the proteins encoded by these genes and the mechanisms of chemotherapy in NSCLC patients. Polymorphisms in the promoter region of these genes regulate protein expression and affect the ability to repair DNA damage (platinum compounds) and cell division (several third generation cytostatics). Because of limited, incomplete or no data on the clinical impact of *ERCC1* (rs11615, rs3212986) and *TOP2A* (rs13695, rs34300454, rs11540720) polymorphisms in NSCLC we decided to examined their influence on the efficiency and toxicity of platinum‐based chemotherapy. The selection of polymorphisms was based on dbSNP data base.

## 2 MATERIALS AND METHODS

### 2.1 Patients

We examined gene polymorphisms in 113 NSCLC Caucasian patients (70% male and 30% female) (Table 1) recruited between 2010 and 2015 at the Medical University of Lublin and the University of Medical Sciences in Poznań. Seventy‐one patients were included in the survival analysis due to the availability of accurate clinical data. All patients received first line chemotherapy based on cisplatin compounds, with was the inclusion criteria allowing participation in the study by patients in NSCLC recurrence after surgical resection or chemoradiotherapy. Clinicopathological data from this group of patients are summarized in Table 2. Treatment regimens of first line chemotherapy used in the studied group are presented in Table 3.

**Table 1:** General characteristics of non‐small cell lung cancer patients

Gender	
Male, n (%)	79 (70)
Female, n (%)	34 (30)
Age in years	
Age median ± SD	65 ± 7.5
≥65, n (%)	64 (56.6)
<65, n (%)	49 (43.4)
Pathomorphological diagnosis	
Adenocarcinoma, n (%)	51 (45.1)
Squamous cell carcinoma, n (%)	57 (50.5)
NOS, n (%)	5 (4.4)

Abbreviations: NOS, NSCLC‐not otherwise specified.

**Table 2:** Clinicopathological characteristics of a group of patients with complete data on the effectiveness of chemotherapy and survival

Studied group (n)	71
Performance status (PS)	
PS = 0, n (%)	10 (14.1)
PS = 1, n (%)	48 (67.6)
PS = 2, n (%)	10 (14.1)
PS = 3, n (%)	3 (4.2)
Disease stage (TNM)	
II‐III, n (%)	39 (55)
IV, n (%)	32 (45)
Smoking status	
Smokers, n (%)	66 (93)
Nonsmokers, n (%)	5 (7)
Weight loss	
<5% (n, %)	32 (45)
≥5% (n, %)	39 (55)
Occupational exposure on carcinogens	
Yes, n (%)	58 (81.7)
No, n (%)	13 (18.3)
Prior surgical treatment	
Yes (n, %)	6 (8.5)
No (n, %)	65 (91.5)
Radiotherapy	
No (n, %)	29 (40.8)
Prior radiochemotherapy	19 (26.8)
Palliative radiotherapy	23 (32.4)
Response to first line platinum‐based chemotherapy	
PD, n (%)	18 (25.4)
SD, n (%)	19 (26.8)
PR, n (%)	29 (40.8)
CR, n (%)	5 (7)
Neutropenia during chemotherapy (any grade)	
Yes, n (%)	38 (53.5)
No, n (%)	33 (46.5)
Anemia during chemotherapy (any grade)	
Yes, n (%)	51 (71.8)
No, n (%)	20 (28.2)

Abbreviations: CR, complete remission; PD, progression disease; PR, partial remission; SD, stable disease.

**Table 3:** Treatment regimens of chemotherapy in the studied group

First line chemotherapy	
Cisplatin or carboplatin and vinorelbine, n (%)	43 (60.6)
Cisplatin or carboplatin and gemcitabine, n (%)	10 (14.1)
Cisplatin and pemetreksed, n (%)	17 (23.9)
Cisplatin and teoposide, n (%)	1 (1.4)
Second‐line chemotherapy	
Cisplatin and vinorelbine, n (%)	1 (1.4)
Cisplatin and gemcitabine, n (%)	2 (2.8)
Carboplatin and paclitaxel, n (%)	1 (1.4)
Carboplatin and docetaxel, n (%)	1 (1.4)
Nivolumab, n (%)	3 (4.2)
Avelumab, n (%)	1 (1.4)
Paclitaxel, n (%)	6 (8.5)
Docetaxel, n (%)	6 (8.5)
Third‐line chemotherapy	
Carboplatin and paclitaxel, n (%)	1 (1.4)
Paclitaxel, n (%)	1 (1.4)
Gemcitabine, n (%)	1 (1.4)
Vinorelbine, n (%)	1 (1.4)

The study protocol was approved by the Committee of Ethics and Research at the Medical University of Lublin (KE‐0254/142/2010). All procedures performed in the studies involving human participants were in accordance with the 1964 Helsinki declaration and its later amendments or comparable ethical standards. Written informed consent was obtained from each patient before participation in this study.

### 2.2 Polymorphism analysis

DNA was isolated from whole blood using the Qiamp DNA Blood Mini kit (Qiagen) according to the manufacturer's instruction. The quality of isolated DNA was analyzed using the BioPhotometer UV/Vis Spectrophotometer (Eppendorf).

We examined five SNPs: rs11615 (*ERCC1*), rs3212986 (*ERCC1*), rs13695 (*TOP2A*), rs34300454 (*TOP2A*), rs11540720 (*TOP2A*) using the qPCR (quantitative real‐time PCR) method. Quantitative real‐time PCR was performed using 6 µL of Genotyping MasterMix (Life Technologies), 1.25 µL of DNase‐Free, RNase‐Free Water, 2 µL of DNA, 0.75 µL of TaqMan SNP Genotyping Assay (Life Technologies). The concentration of template in the qPCR reaction was 5 ng/µL. The sequences of the primers and TaqMan probe used were ordered as ready kits from Thermo Fisher, all information on these kits is available on the manufacturer's website. Reactions were performed in the following conditions: initial denaturation and enzyme activation: 95°C for 10 minutes, 40 cycles: 95°C for 15 seconds, and 62°C for 90 seconds on an Eco Illumina Real‐Time PCR system device (Illumina Inc).

### 2.3 Statistical analysis

Survival analysis includes Cox regression (proportional hazards model) and Kaplan‐Meier survival analysis, and was performed with the use of software (Belgium). Clinical and demographic factors were analyzed using the Fisher Chi square test. *P*‐values below .05 were considered as significant.

## 3 RESULTS

### 3.1 The frequency of different genotypes occurrence

In the whole group of NSCLC patients C/C genotypes we observed in 65 patients (57.5%) in SNP rs13695, in 86th (76.1%), in SNP rs34300454, and in 109 patients (96.5%) in SNP rs1154720 of *TOP2A* gene. T/T homozygotes in these polymorphic sites were very rare or absent. Fifty‐three of patients (56.9%) showed the A/A genotype and 60 patients (53.1%) carried the A/G genotype of rs11615 *ERCC1* gene. In 73 patients (64.6%), we observed the C/C genotype of rs3212986 *ERCC1* gene, and the A/A homozygous variant was only present in single NSCLC patients. The frequency of individual genotypes did not differ significantly between the whole group of patients and the group of patients treated with chemotherapy. The frequency of particular genotypes in *TOP2A* and *ERCC1* genes is presented in Table 4.

**Table 4:** The frequency of particular genotypes in *TOP2A* and *ERCC1* genes

Gene	SNP ID	Genotype	Genotype frequency in whole group of NSCLC patients, n (%)	Genotype frequency in group of NSCLC patients treated with chemotherapy, n (%)
*TOP2A*	rs13695	C/C	65 (57.5%)	39 (55%)
		T/T	4 (3.6%)	4 (5.6%)
		C/T	44 (38.9%)	28 (39.4%)
	rs34300454	C/C	86 (76.1%)	48 (67.6%)
		T/T	0 (0)	0 (0)
		C/T	27 (23.9%)	23 (32.4%)
	rs11540720	C/C	109 (96.5%)	69 (97.2%)
		T/T	0 (0)	0 (0)
		C/T	4 (3.5%)	2 (2.8)
*ERCC1*	rs11615	A/A	53 (46.9%)	37 (52.1%)
		G/G	0 (0)	0 (0)
		A/G	60 (53.1%)	34 (47.9%)
	rs3212986	A/A	4 (3.5%)	1 (1.4%)
		C/C	73 (64.6%)	50 (70.4%)
		A/C	36 (31.9%)	20 (28.2%)

Abbreviations: NSCLC, non‐small cell lung cancer; SNP, single nucleotide polymorphism.

We evaluated the association between genotypes and demographic and clinical factors in NSCLC patients. We did not find any significant relationship (*P* > .05) between the genotype of the *TOP2A* and *ERCC1* genes and age, gender, disease stage, pathomorphological diagnosis (non‐squamous cell carcinoma vs. squamous cell carcinoma) or exposure to carcinogens (occupational or smoking status). The incidence of individual polymorphisms in patients with squamous cell carcinoma and in patients with adenocarcinoma was similar in all groups studied, slight differences were not statistically significant. The lack of such relationships occurred both in the whole group of patients and in the group of patients treated with chemotherapy.

### 3.2 SNPs and response to first line chemotherapy

In the studied group, 7% of patients achieved complete remission (CR) as a result of first line chemotherapy. Control of the disease was observed in 74.6% of patients, of which partial remission and stable disease occurred in 40.8% and 26.8% of patients respectively. Progression of disease was observed in 25.4% of patients. Response to the treatment was not dependent on the presence of particular polymorphisms in the *ERCC1* and *TOP2A* genes.

### 3.3 Clinical and genetic factors and median progression free survival after first line chemotherapy

Median progression free survival (mPFS) for the studied population was 6 months. In patients who had prior surgery treatment mPFS was significantly longer compared to other patients (30 months vs 5 months; *P* = .0022; *χ* ^2^ = 9.36; hazard ratio (HR) = 0.3221; 95% CI: 0.1559‐0.6656). Likewise, in patients in stage IIIA or IIIB of the disease PFS was longer compared to patients in stage IV (8.5 months vs 3.5 months; *P* = .0003; *χ* ^2^ = 13.23; HR = 0.3118; 95% CI: 0.1664‐0.5842).

**Figure 1:**
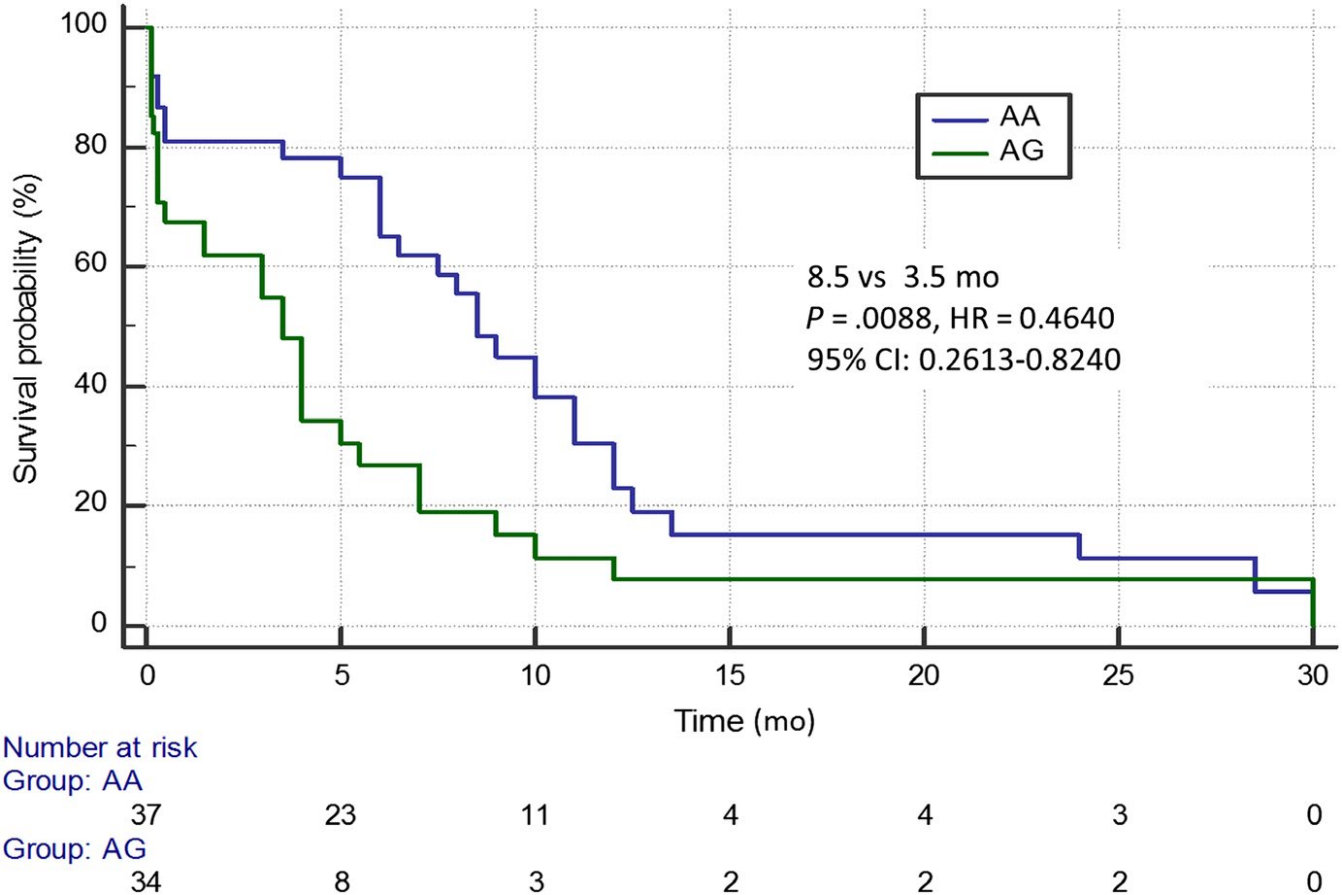
Progression free survival of non‐small cell lung cancer patients with different genotypes of rs11615 *ERCC1* gene estimated in Kaplan‐Meier method

**Figure 2:**
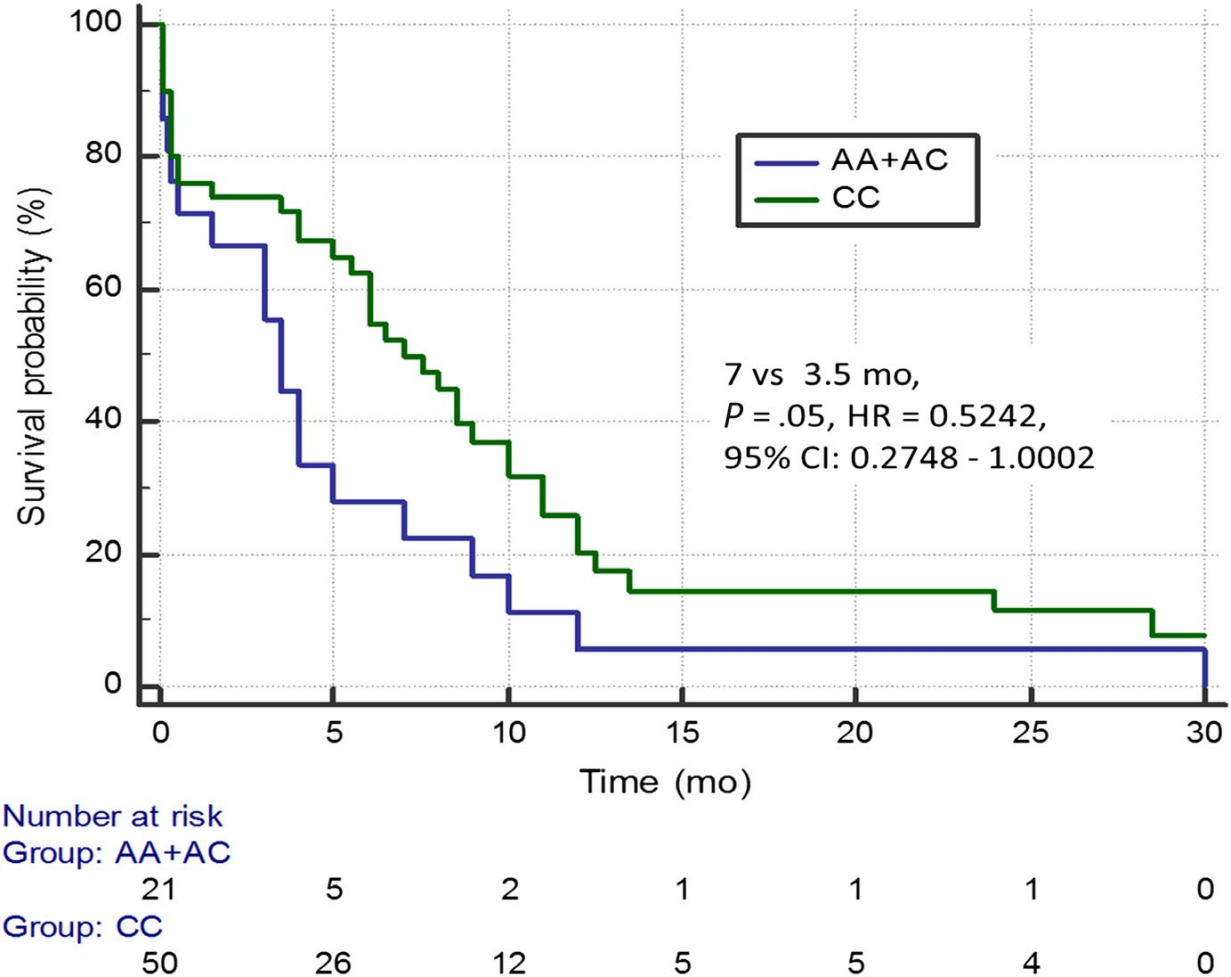
Progression free survival of non‐small cell lung cancer patients with different genotypes of rs3212986 *ERCC1* gene estimated in Kaplan‐Meier method

**Figure 3:**
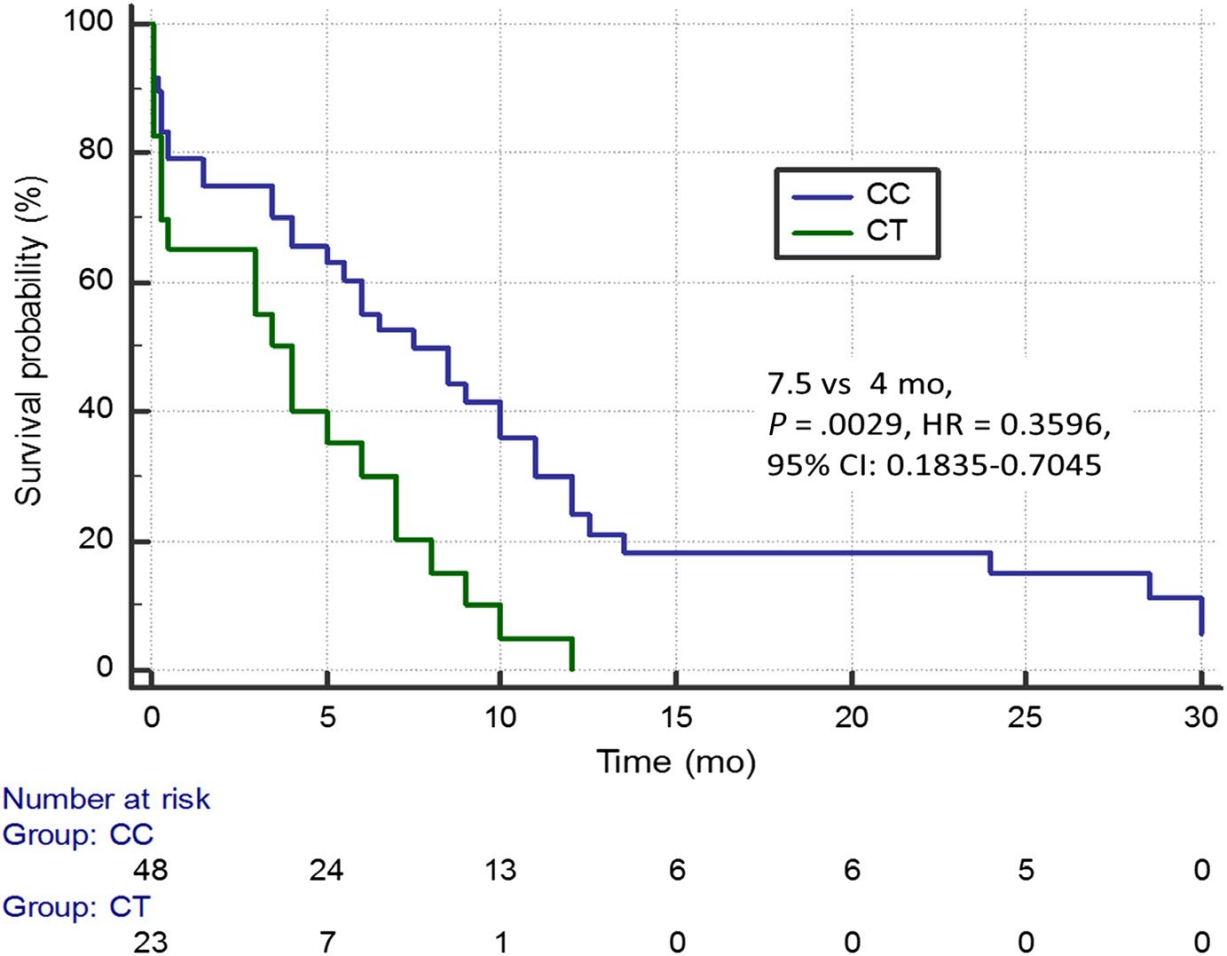
Progression free survival of non‐small cell lung cancer patients with different genotypes of rs34300454 *TOP2A* gene estimated in Kaplan‐Meier method

Patients with the A/A genotype of the rs11615 *ERCC1* gene had significantly higher median PFS than patients with the A/G genotype (8.5 months vs 3.5 months; *P* = .0088; *χ* ^2^ = 8.86; HR = 0.4640; 95% CI: 0.2613‐0.8240; Figure 1). In addition, carriers of the C/C genotype of rs3212986 *ERCC1* gene demonstrated longer PFS compared to patients with other variants of this polymorphism (7 months vs 3.5 months; *P* = .05; *χ* ^2^ = 3.8387; HR = 0.5242; 95% CI: 0.2748‐1.0002; Figure 2). Patients with the C/C genotype of the rs34300454 *TOP2A* gene showed significantly higher mPFS than carriers of the C/T genotype (7.5 months vs 4 months; *P* = .0029; *χ* ^2^ = 8.8858; HR = 0.3596; 95% CI: 0.1835‐0.7045; Figure 3).

Other demographic, clinical and genetic factors had no statistically significant effect on the PFS duration.

### 3.4 Clinical and genetic factors and median overall survival after first line chemotherapy

In the chemotherapy‐treated population, the median overall survival (mOS) was 12 months. Patients undergoing previous surgical treatment had significantly higher mOS compared to patients without surgical treatment (32 months vs 12 months; *P* = .0056; *χ* ^2^ = 7.6871; HR = 0.3710; 95% CI: 0.1841‐0.7478). In patients who previously underwent radiochemotherapy, OS was significantly longer compared to patients who did not undergo radiochemotherapy (14.5 months vs 12 months; *P* = .05, *χ* ^2^ = 3.8151; HR = 0.5627; 95% CI: 0.3160‐1.0020). In patients in stage IIIA or IIIB of the disease mOS was higher than in metastatic patients (16.5 months vs 7 months; *P* = .0037; *χ* ^2^ = 8.4469; HR = 0.4097; 95% CI: 0.2245‐0.7479).

**Figure 4:**
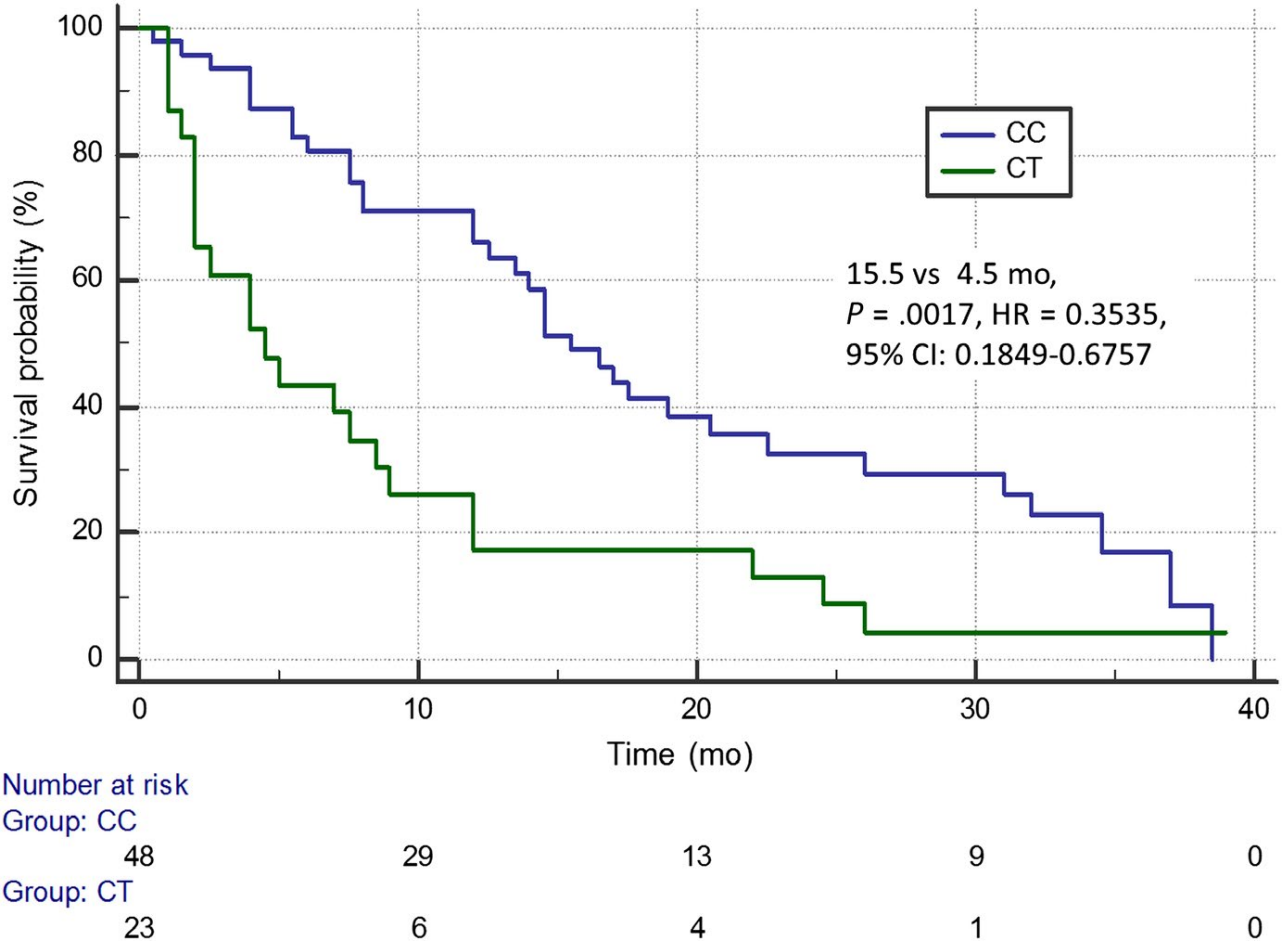
Overall survival of non‐small cell lung cancer patients with different genotypes of rs34300454 *TOP2A* gene estimated in Kaplan‐Meier method

In addition, carriers of the C/C genotype of rs34300454 *TOP2A* gene demonstrated significantly longer OS compared with patients with the C/T genotype (15.5 months vs 4.5 months; *P* = .0017; *χ* ^2^ = 9.8962; HR = 0.3535; 95% CI: 0.1849‐0.6757; Figure 4). There were no statistically significant differences in the duration of OS in patients differing in terms of demographic, clinical and genetic factors.

### 3.5 Survival analysis by multivariate Cox logistic regression

Multivariate Cox logistic regression confirmed that the strongest factors that increased the risk of progression in NSCLC patients treated with platinum‐based chemotherapy were: lack of previous surgical treatment, hematological complications (especially 3rd and 4th grade, according to the NCI‐CTC criteria), advanced disease stage, A/G genotype of rs11615 *ERCC1* gene, and C/T genotype of rs34300454 *TOP2A* gene. According to the Cox regression model, demographic, clinical and genetic factors that increased the risk of death were: lack of second line chemotherapy, lack of occupational exposure to carcinogens, lack of previous chemoradiotherapy and lack of prior surgical treatment, severe hematological complications, negative smoking status, C/T genotype of rs13695 *TOP2A* gene, and C/T genotype of rs34300454 *TOP2A* gene (Table 5). The positive effects of exposure to carcinogens and smoking on the reduction of the risk of death can be explained by the higher effectiveness of immunotherapy in this group of patients. Four patients (5.6%) benefited from nivolumab or avelumab therapy in the second line of treatment in the group of patients treated with first line chemotherapy.

**Table 5:** The multivariate Cox logistic regression of factors affecting the progression free survival and overall survival in NSCLC patients treated with first line chemotherapy

Factor	*P* value	Hazard ratio	95% CI	
Progression free survival				
No surgical treatment	.0051	9.1637	1.9449‐43.1752	
Severe hematological complications	.0160	2.3047	1.1685‐4.5457	
Metastatic disease	.0095	2.2547	1.2200‐4.1669	
A/G genotype of rs11615 *ERCC1* gene	.0404	1.9193	1.0289‐3.5801	
C/T genotype of rs34300454 *TOP2A* gene	.0006	2.8826	1.5792‐5.2617	
Overall model fit: *P* < .0001, *χ* ^2^ = 42 052				
Overall survival				
Lack of second line chemotherapy	<.0001	5.9322		2.8476‐12.3584
Occupational exposure on carcinogens	.0207	0.4177		0.1994‐0.8751
No surgical treatment	<.0001	35.0140		7.7403‐158.3883
Severe hematological complications	.0020	3.8917		1.6458‐9.2024
Lack of radiotherapy	.0023	2.9246		1.4686‐5.8244
Positive smoking status	.0037	0.1580		0.0454‐0.5502
C/T genotype of rs13695 *TOP2A*	.0457	1.6729		1.0098‐2.7713
rs34300454 *TOP2A*	.0008	2.8946		1.5598‐5.3713
Overall model fit: *P* < .0001, *χ* ^2^ = 58 230				

Abbreviation: NSCLC, non‐small cell lung cancer.

### 3.6 SNPs and hematological toxicity

**Figure 5:**
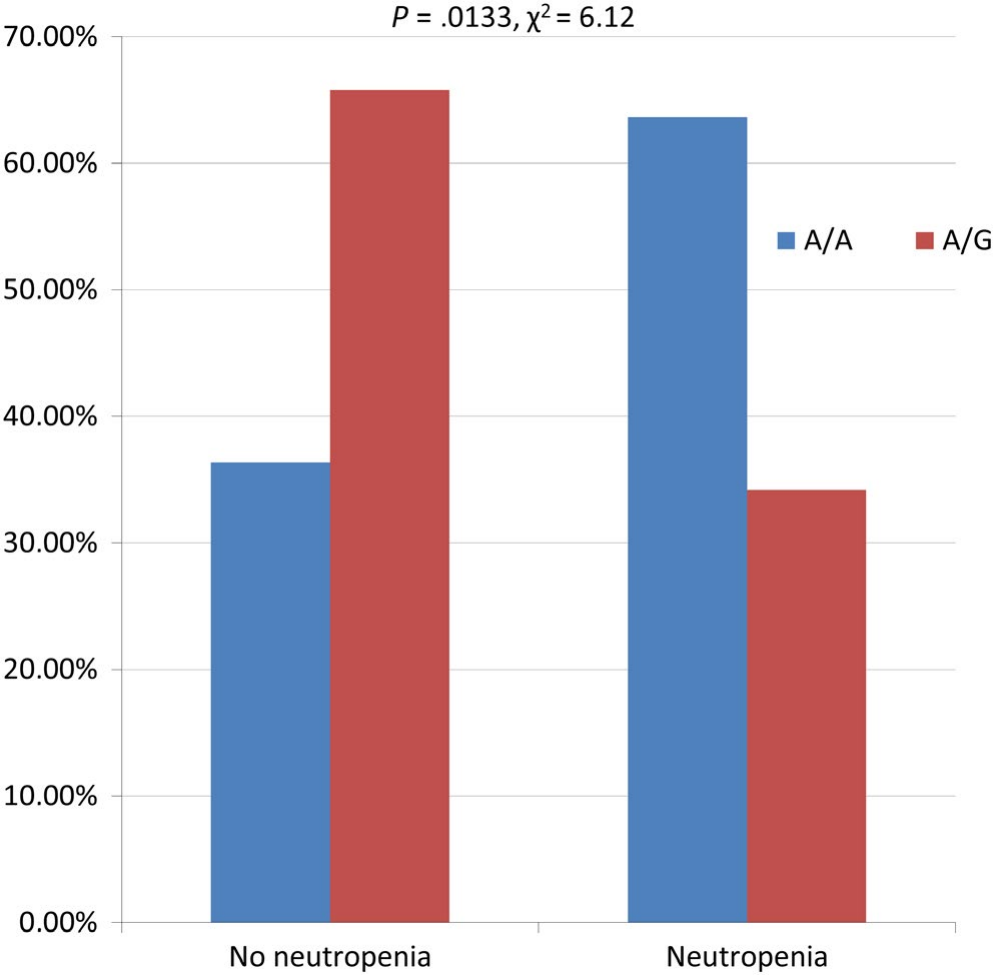
The impact of genotype of rs11615 *ERCC1* on the risk of neutropenia during first line chemotherapy in patients with non‐small cell lung cancer

Patients harboring homozygous A/A genotype in rs11615 of *ERCC1* showed significantly higher risk of neutropenia (any grade) during chemotherapy than heterozygous patients A/G (*P* = .0133; *χ* ^2^ = 6.12; Figure 5). No statistically significant association between the other examined SNPs and chemotherapy toxicities was found.

## 4 DISCUSSION

The occurrence of SNPs in coding or noncoding sequences (mainly promoter) of different genes may have determined changes in the expression, structure and function of various proteins.^20^ Our study describes the results of association between genetic polymorphisms and the outcomes of first line chemotherapy in 71 Polish NSCLC patients. We examined five SNPs. Two are functionally connected with *ERCC1* gene (rs11615, rs3212986) and three with *TOP2A* gene (rs13695, rs34300454, rs11540720).

Sun et al examined 72 patients with NSCLC receiving platinum‐based adjuvant chemotherapy. Their results confirmed the correlation between the high expression of mRNA for the *ERCC1* gene and poor efficacy of chemotherapy.^21^ The association of ERCC1 expression with the effectiveness of chemotherapy could be demonstrated not only at the mRNA level but also at the protein level. The International Adjuvant Lung Cancer Trial (IALT) analysis showed that patients with ERCC1 protein negative tumors have better adjuvant cisplatin‐based chemotherapy results compared to patients with ERCC1 protein positive tumors.^22^

Previous genetic studies had shown that rs3212986 and rs11615 *ERCC1* gene polymorphisms may be a predictive factor for chemotherapy in NSCLC patients. Moreover, some polymorphic variants of these SNPs may affect the risk of lung cancer.^23^, ^24^, ^25^, ^26^, ^27^, ^28^ Yu et al revealed that NSCLC patients carrying the A/A genotype in rs3212986 of *ERCC1* gene had a higher risk of lung cancer, especially in the smoking population.^28^ Zhou et al observed that rs3212986 *ERCC1* gene polymorphism may be a useful predictor of OS in advanced NSCLC patients treated with platinum‐based chemotherapy. In this study, the median survival time was 22.3 months in patients with the C/C genotype and 13.4 months in patients with the C/A or A/A genotypes, suggesting that the presence of the A allele was associated with a poor outcome.^23^

Takenaka et al examined both SNPs in the *ERCC1* gene in 122 Japanese NSCLC patients who underwent a complete resection. They noted that the patients with the C/A genotype of rs3212986 *ERCC1* gene showed significantly poorer disease‐free survival (DFS) and OS than those with the C/C genotype. In addition, no relationship was observed between different variants of rs11615 *ERCC1* gene and DFS or OS. These two SNPs did not correlate with either ERCC1 protein expression or platinum sensitivity.^27^

Isla et al noticed that patients with advanced NSCLC treated with cisplatin‐based chemotherapy with the G/G genotype of rs11615 *ERCC1* gene achieved better OS.^24^ The role of genetic polymorphisms of the *ERCC1* gene in advanced NSCLC patients was evaluated in a meta‐analysis by Dong et al and Wei et al. The authors demonstrated that NSCLC patients with the G/G or A/G genotype of rs11615 *ERCC1* gene exhibited a higher probability of response to platinum‐based treatment than those with the A/A genotype.^29^, ^30^ Other three meta‐analyses confirmed these observations in advanced NSCLC patients treated with platinum‐based chemotherapy. Tan et al found an association between the A allele of rs11615 *ERCC1* gene and shorter OS.^31^ Similar effect was shown by Yang et al and Xu et al. They shown shorter OS in patients with the A allele of rs11615 *ERCC1* gene.^32^, ^33^ Li et al conducted a study to investigate the gene polymorphisms in the prognosis of gastric cancer. They found that the A/A genotype in rs11615 of ERCC1 was associated with higher risk of death from gastric cancer, and there was no association between ERCC1 rs3212986 polymorphism and prognosis of gastric cancer.^34^ Another study found that A/A genotype in rs11615 of *ERCC1* were associated with longer survival time in gastric cancer patients.^35^ Chen et al indicates the patients carrying A/A + G/A genotypes in rs11615 of *ERCC1* are at higher risk of endometrial carcinoma than those with G/G genotype. Homozygous G/G genotype in rs11615 of *ERCC1* had a higher response rate of chemotherapy than patients with G/A + A/A genotype.^36^

Our study only partially confirmed these observations. We found no relationship between the presence of individual polymorphic variants and the occurrence of a response to treatment. Homozygous C/C genotype of rs3212986 in the *ERCC1* gene showed a favorable prognostic value and was associated with significant prolongation of PFS. However, the homozygous A/A genotype of rs11615 *ERCC1* gene seems to have a beneficial effect on PFS and OS in patients undergoing chemotherapy with platinum compounds.

Dingemans et al found that the high expression of TOP2A in tumor tissue was a poor prognostic factor compared to the low or intermediate expression of TOP2A in NSCLC patients. The authors suggested that high TOP2A expression was associated with cancer cell resistance to chemotherapy.^37^ Some previous clinical studies reported that there was no significant association between TOP2A expression and clinical parameters in NSCLC patients.^38^ However, Hou et al reported high expression of TOP2A significantly increased risk of death in NSCLC patients, especially in lung adenocarcinoma patients. Moreover, they showed that high expression of TOP2A was associated with metastases in lymph nodes, higher disease stage and smoking status. These results support the conclusion that TOP2A was associated with worse prognosis in NSCLC patients.^39^ Only one publication deals with the SNPs in the *TOP2A* gene in NSCLC patients. Wang et al showed that rs471692 *TOP2A* gene SNPs were not associated with the response to chemoradiotherapy in NSCLC patients.^40^

To our knowledge, this current study is the first clinical report suggesting that homozygous C/C genotypes of rs34300454 and rs13695 *TOP2A* gene showed a favorable prognostic value and they could be associated with significant prolongation of OS and/or PFS in NSCLC patients treated with first line chemotherapy. The only publication that describes polymorphism of rs13695 *TOP2A* gene concerns breast cancer patients. The authors found that there was an association between rs13695 genotype and *TOP2A* gene expression.^41^

In our patients, hematological toxicity induced by chemotherapy was associated with rs11615 *ERCC1* gene polymorphism. Particularly, the AA genotype of this SNP was associated with higher risk of neutropenia. No effect of other polymorphisms on hematological toxicity was found. Perez‐Ramirez et al showed a correlation between the occurrence of the A allele of rs11615 *ERCC1* gene and higher risk of general toxicity for platinum‐based chemotherapy in NSCLC patients, but they did not find the influence of this SNP on hematological toxicity.^42^

## 5 CONCLUSIONS

In conclusion, it is important to create an effective treatment plan which will be tailored to an individual patient. We would like to note that we, for the first time worldwide, indicated that genotypes of rs34300454 and rs13695 *TOP2A* gene showed a favorable prognostic value in NSCLC patients treated with platinum‐based chemotherapy. We also showed that a significant risk of hematological complications is moderated by rs11615 of the *ERCC1* gene. The results of these studies shows that the scoring of polymorphisms may be useful as a prognostic factors in chemotherapy‐treated patients with advanced NSCLC. Exploratory findings should be verified in independent, larger populations.

## CONFLICT OF INTEREST

None declared.

## Data Availability

The data that support the findings of this study are available from the corresponding author upon reasonable request.
